# Acute and Chronic Health Risk Assessment for Automobile Users Due to Inhalation Exposure to Volatile Organic Compounds and Carbonyl Compounds

**DOI:** 10.3390/toxics12120843

**Published:** 2024-11-24

**Authors:** Jeong-In Jeon, Eun-Ju Lim, Young-Jun Byun, Min-Kwang Kim, Hyun-Woo Lee, Cha-Ryung Kim, In-Ji Park, Ho-Hyun Kim, Cheol-Min Lee

**Affiliations:** 1 Department of Chemical and Environmental Engineering, Seokyeong University, Seoul 02713, Republic of Korea; hhzz01@skuniv.ac.kr (J.-I.J.); ho04sh@skuniv.ac.kr (H.-H.K.); 2 Korea Testing Laboratory, Seoul 08389, Republic of Korea; limeju@ktl.re.kr (E.-J.L.); younghost@ktl.re.kr (Y.-J.B.); kmk9404@ktl.re.kr (M.-K.K.); 3 Korea Transportation Safety Authority, Hwaseong 18247, Republic of Korea; peterlee@kotsa.or.kr (H.-W.L.); cha1052@kotsa.or.kr (C.-R.K.); coolinji@kotsa.or.kr (I.-J.P.)

**Keywords:** automobiles, indoor air quality, volatile organic compounds, carbonyl compounds, health risk assessment

## Abstract

Since automobiles are the primary means of transportation in modern society, the assessment of health effects from acute and chronic exposure to pollutants in automobiles is crucial. In this study, the concentration of volatile organic compounds (VOCs), carbonyl compounds, and odor-including substances in newly manufactured automobiles were analyzed, and exposure factors reflecting automobile user characteristics were selected for health risk assessment. Toluene exhibited the highest concentration (203.5 ± 379.3 μg/m^3^) among all measured pollutants. The acute and chronic non-carcinogenic health risk assessments indicated that the pollutants did not exceed their standards in any of the automobiles, suggesting no immediate health risks. However, in the chronic carcinogenic health risk assessment, acrylonitrile exceeded its standard value in all automobiles, indicating potential carcinogenic effects from long-term exposure. The findings suggest that careful estimation of lifetime exposure effects to pollutants inside new automobiles is required. Future studies should investigate specific pollutants generated by automotive materials and manufacturing processes to identify pollutant sources and reduce health risks to automobile users.

## 1. Introduction

Indoor air quality (IAQ) is a critical factor in assessing occupant health risks, as people spend over 80% of their day in indoor environments. The World Health Organization (WHO) reports that indoor air pollution is responsible for 3.8 million deaths annually [1]. According to the Ministry of Land, Infrastructure, and Transport (MOLIT) statistics system and big data platform, the number of registered automobiles in Korea has increased by 2–3% annually since 2013, with the total reaching 25,461,361 as of November 2022 [2]. Considering the total population of the country (51,450,829), this indicates that each individual owns 0.49 vehicles. As automobiles serve as the primary means of transportation in contemporary society, IAQ concerns extend to the indoor environment of automobiles. In particular, “new car syndrome”, which refers to symptoms experienced by drivers and passengers in automotive cabins (e.g., fatigue, headache, and eye and skin irritation), has emerged as significant IAQ problem in automobiles [3].

Exposure to various pollutants in vehicles has been a major concern for several decades. Pollutants in vehicles originate from car interior materials, fuel leaks, exhaust gas leakage, and infiltration from the outside environment. According to the survey results on worker mobility behavior conducted by Statistics Korea in 2023, people spend an average of 72.6 min commuting to and from work [4]. Americans spend 5.5% of their time in automobiles [5], while this value is 3.2% for Canadians [6]. In Italy, people spend 10% of their day inside vehicles [7]. Although the time spent in automobiles during the day is minimal, automobiles are used almost daily for commuting. Therefore, it is necessary to consider the health effects of both short-term acute exposure and long-term chronic exposure to pollutants inside vehicles.

Automobile manufacturers have undertaken voluntary efforts to improve vehicle IAQ. They have developed standards to examine harmful substances emitted from car interior materials and parts before the final manufacturing stage and to evaluate odor compounds that may adversely affect customer satisfaction [8]. The smell in automotive cabins delivers the first impression of vehicles to consumers [8]. Since such odors may cause respiratory hypersensitivity and mental stress for passengers, managing automotive cabin odors is imperative [9].

Air pollution in automotive cabins primarily results from volatile organic compounds (VOCs) and carbonyl compounds emitted from interior materials of newly manufactured automobiles, pollutants generated by user activities and exhaust gas, and infiltration of surrounding air pollutants due to traffic conditions. The unique smell in new cars is attributed to VOCs [10], which can adversely affect the respiratory system, nervous system, and skin [10]. Various interior materials (e.g., sheets, artificial leather, and internal covers) in automotive cabins and their associated pollutants are the main causes of new car syndrome. These interior materials are found in various products, such as plastic materials, adhesives, paints, and thinners, and are available in multiple forms [11,12,13,14]. Additionally, fine particulate matter, another pollutant in automobiles mainly generated by incomplete combustion of fossil fuels, enters automotive cabins through windows and external air circulation during operation. This exposure affects all vehicle occupants, as pollution from outdoor sources such as traffic and industry enter the vehicle interior environment [15].

Among the various pollutants in automotive cabins, VOCs and carbonyl compounds are present in high concentrations, and exposure to them can lead to adverse health effects ranging from allergies to serious chronic diseases, including cancer [16]. VOCs, having a boiling point below 250 °C at standard atmospheric pressure (101.325 kPa), exist as gases in the atmosphere and contribute significantly to ozone and microparticle formation through photochemical reactions under certain conditions. VOC exposure can cause acute symptoms such as nose, neck, and eye irritation; headache; nausea; dizziness; and allergic skin reactions. They may also damage internal organs, including the liver and kidneys. Regarding chronic health risks, VOCs can cause sensory disorders in the nervous system, visual and auditory impairments, memory loss, sleep disorders, emotional disorders, and mental disorders (e.g., fatigue). Notably, some VOCs, such as benzene, 1,3-butadiene, vinyl chloride, and formaldehyde, are classified as human carcinogens (Group 1) by the International Agency for Research on Cancer (IARC) [17]. Acetaldehyde, ethyl benzene, and styrene are classified as Group 2 carcinogens by IARC [17].

This study examines the health effects of pollutant exposure in automobiles by utilizing exposure factors that reflect user characteristics. We measured and analyzed the concentrations of VOCs, carbonyl compounds, and odor-inducing substances inside newly manufactured automobiles. We selected exposure factors reflecting automobile user characteristics for exposure and risk assessment. Furthermore, we compared health risks from acute and chronic exposure by conducting comprehensive health risk assessments that consider the environmental characteristics of automobiles.

## 2. Materials and Methods

### 2.1. Sample Collection and Analysis

In this study, the IAQ inside automobiles was analyzed by selecting pollutants commonly present in new cars as target pollutants: 61 VOCs, 13 carbonyl compounds, and 6 odor substances.

The IAQ measurements were performed for four imported vehicles and four domestic vehicles from April to August 2023. The target pollutants were measured at 25 °C and 50% relative humidity in accordance with MOLIT test environment specifications.

For VOCs and carbonyl compounds, sample collection and analysis were conducted following the IAQ measurement method in Annex 2 of the “Indoor Air Quality Management Standards for Newly Manufactured Automobiles” announced by MOLIT [18] and the “Air Pollution Test Standards” [19], “Indoor Air Quality Test Standards” [20], and “Odor Test Standards” [21] of the National Institute of Environmental Research (NIER).

VOCs were collected using an MP-∑30 KN air sampler (SIBATA Scientific Technology Ltd., Tokyo, Japan) with an adsorption tube filled with Tenax TA at a flow rate of 100 mL/min for approximately 20 min. The collected samples were thermally desorbed using a thermal desorption device and quantified using gas chromatography–flame ionization detection (GC/FID). Carbonyl compounds were measured using a DNPH derivatization cartridge at a flow rate of 1 L/min for approximately 20 min, with an ozone scrubber to minimize ozone interference. The DNPH cartridge samples were extracted using acetonitrile solvent, and the extracts were analyzed using high-performance liquid chromatography (HPLC) with a 360 nm UV detector. Ammonia was measured and analyzed using boric acid solution absorption ultraviolet/visible spectroscopy. Using an absorption collection vessel and LV-40BR (SIBATA Scientific Technology Ltd., Japan), the odorous air was collected into a 0.5% boric acid solution, and phenol-sodium nitroprusside solution and sodium hypochlorite solution were added to the analysis sample, followed by measurement at 640 nm using a spectrophotometer (Agilent Cary 3500, Agilent, Santa Clara, California, USA). Sulfur compounds were analyzed using the low-temperature condensation GC/FID method, and trimethylamine was analyzed using the solid-phase micro-extraction (SPME) method with GC/FID.

### 2.2. Quality Assurance and Control

Quality assurance/quality control (QA/QC) of measurement and analysis was performed to verify the reliability of the analysis results. Table S1 presents the quality control results. The reproducibility of the analytical instrument was evaluated by repeatedly analyzing the standard material and obtaining the retention time (RT). The method detection limit (MDL) was calculated by analyzing the concentrations of the target pollutants expected to reach MDL seven times and then multiplying the standard deviation (SD) for the measured concentration of each pollutant by 3.14.

### 2.3. Health Risk Assessment

A health risk assessment was conducted on inhalation exposure to the VOCs, carbonyl compounds, and odor substances emitted from automotive interior materials, considering automobile user characteristics. The health risk assessment was performed for both acute and chronic exposure. Table S2 presents the toxicity information for acute and chronic exposure to the target pollutants used in this study.

#### 2.3.1. Acute Health Risk Assessment

Pollutants present at high concentrations inside automobiles can have adverse health effects in humans during short-term exposure. The main symptoms include headache, dizziness, shortness of breath, and eye or skin irritation. In acute health risk assessment, exposure to high concentrations of pollutants over short periods is considered. The health risk was calculated using the following equation:

(1) $${HQ}_{acut\;e}=\frac{C_{max}(\mu g/m^{3})}{R\;EL(\mu g/m^{3})}$$

where HQ_acute_ is the acute health risk and C_max_ is the maximum concentration of the pollutants inside the vehicle (μg/m^3^), and the reference exposure level (REL) is the acute inhalation reference concentration (μg/m^3^). Health risks were considered possible when HQ_acute_ exceeded 1.

#### 2.3.2. Chronic Health Risk Assessment

Chronic health risk assessment was conducted for carcinogens and non-carcinogens, with consideration of automobile user exposure. The risk was evaluated by calculating the excess cancer risk (ECR) for carcinogens and the hazard quotient (HQ) for non-carcinogens. ECR was determined by multiplying the cancer slope factor (CSF) by the lifetime average daily dose (LADD), while HQ was calculated by dividing the average daily dose (ADD) by the reference dose (RfD). The CSF was converted to incorporate the unit risk (UR), body weight (BW), and inhalation rate (IR), and RfD was converted to account for the reference concentration (RfC), BW, and IR (Equations (2) and (3), respectively).

(2) $$CSF((mg/kg/day{)}^{-1})=\frac{UR((\mu g/m^{3}{)}^{-1})\times BW(kg)}{IR(m^{3}/day)}\times \frac{1000\;\mu g}{1\;mg}$$

(3) $$RfD(mg/kg/day)=\frac{RfC(mg/m^{3})\times IR(m^{3}/day)}{BW(kg)}$$

For carcinogenic risk assessment, lifetime exposure effects were considered, and LADD was calculated using life expectancy (Equation (4)). For non-carcinogenic risk assessment, ADD was calculated using Equation (5). Both calculations assumed 100% absorption of exposure pollutants by the human body.

(4) $$LADD=\frac{C\times IR\times EF\times ED\times ET}{BW\times LT}$$

(5) $$ADD=\frac{C\times IR\times EF\times ED\times ET}{BW\times AT}$$

Here, LADD is the lifetime average daily dose (mg/kg/day), C is the pollutant concentration (mg/m^3^), IR is the inhalation rate (m^3^/day), EF is the exposure frequency (day/years), ED is the exposure duration (years), ET is the exposure time, BW is the body weight (kg), LT is the lifetime (day), ADD is the average daily dose (mg/kg/day), and AT is the average time (day).

The exposure factors and data sources are presented in Table S3. The BW and IR factors were obtained from the exposure factor handbook of Koreans published by NIER [22]. The exposure frequency and exposure time were derived from a survey of 1120 automobile drivers conducted between September to October 2023. Based on data from the Korea Auto Dismantlement Recycling Association (KADRA), the average vehicle scrapping cycle was 15.6 years as of 2020; therefore, the exposure duration was set at 15 years [23].

Carcinogenic risk was evaluated using ECR. Health risks were considered possible when the excess cancer probability of one person per million people, which is the naturally occurring risk level specified by the US EPA, exceeded 1.00 × 10^−6^. For non-carcinogenic risk assessment, health risks were considered possible when HQ_chronic_ exceeded 1.

(6) $${ECR}_{chronic}=CSF\times LADD$$

(7) $${HQ}_{chronic}=\frac{ADD}{RfD}$$

## 3. Results

### 3.1. Automotive Pollutant Concentration Distribution

**Figure 1:**
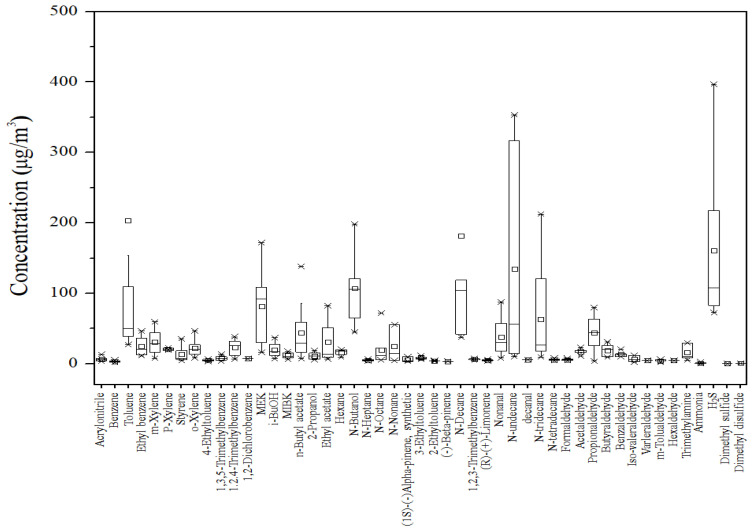
Pollutant concentration distribution in automobiles.

The concentrations and detection rates of VOCs and carbonyl compounds for the eight vehicles are shown in Table S4 and Figure 1. Of the 61 target VOCs, 38 VOCs were detected, while 24 VOCs were present below the detection limit. Among the 13 carbonyl compounds, 10 were detected and 3 were below the detection limit. Toluene exhibited the highest concentration (203.5 ± 379.3 μg/m^3^) among all pollutants, consistent with previous studies measuring VOCs in automobiles [24,25,26].

**Figure 2:**
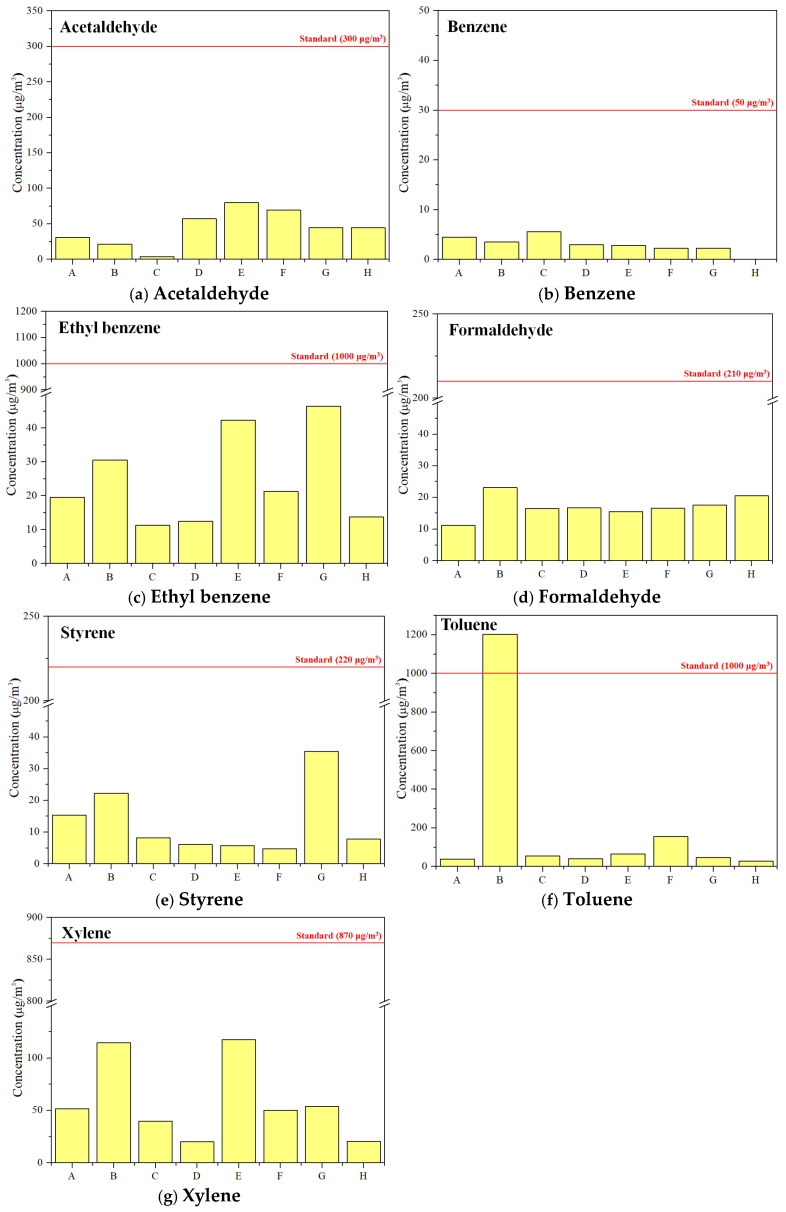
Concentration of the eight pollutants in the vehicles as per the newly manufactured automobile management standards.

Benzene, toluene, xylene, ethyl benzene, styrene, acrolein, acetaldehyde, and formaldehyde, which are the most studied pollutants in vehicles and are set as recommended standards by the MOLIT notice, are examined in detail. Figure 2 shows their concentration distributions. According to the “Indoor Air Quality Management Standards for Newly Manufactured Automobiles”, the recommended standards for these compounds are 30, 1000, 870, 1000, 220, 50, 300, and 210 μg/m³, respectively. Only toluene in vehicle B (1202 μg/m^3^) exceeded the recommended standard. Acrolein concentrations were below the detection limit in all vehicles.

### 3.2. Acute Health Risk Assessment

HQ_acute_ was calculated for pollutants with acute REL values established by the California Office of Environmental Health Hazard Assessment (OEHHA). Methylene chloride, chloroform, carbon tetrachloride, tetrachloride, and acrolein were excluded from the analysis due to concentrations below detection limits in all vehicles. The acute risk assessment results are presented in Table 1.

The calculated HQ_acute_ values for all pollutants remained below 1 across all vehicles, indicating no significant acute health risks. Formaldehyde demonstrated the highest HQ_acute_ values across vehicles: A (2.03 × 10^−1^), B (4.20 × 10^−1^), C (3.00 × 10^−1^), D (3.04 × 10^−1^), E (2.82 × 10^−1^), F (3.01 × 10^−1^), G (3.19 × 10^−1^), and H (3.73 × 10^−1^).

### 3.3. Chronic Health Risk Assessment

#### 3.3.1. Carcinogens

**Figure 3:**
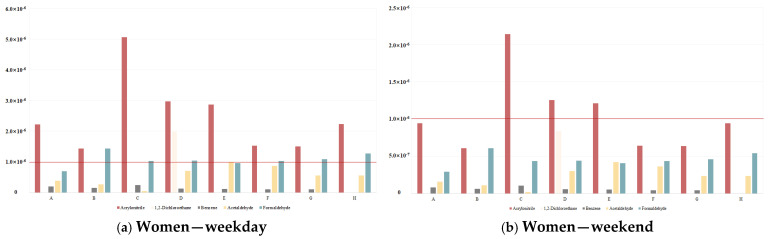

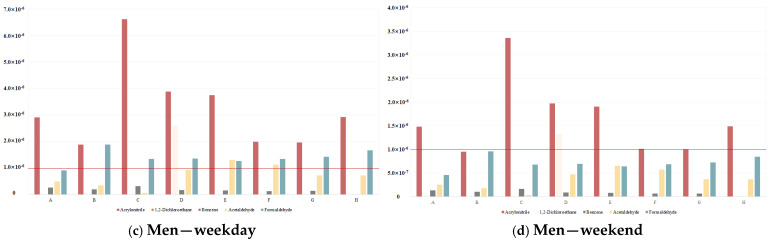
Chronic health risk assessment results for carcinogens.

ECR_chronic_ was calculated for 13 carcinogenic pollutants: acrylonitrile, methylene chloride, chloroform, 1,2,-dichloroethane, benzene, carbon tetrachloride, trichloroethylene, 1,1,2-trichloroethane, 1,2-dibromoethane, tetrachloroethylene, hexachloro-1,3-butadiene, formaldehyde, and acetaldehyde. The analysis focused on five pollutants (acrylonitrile, 1,2-dichloroethane, benzene, acetaldehyde, and formaldehyde) that exhibited both carcinogenic toxicity values and detectable concentrations. Risk calculations were stratified by sex and day of the week, with results presented in Table S5 and Figure 3.

The results indicate that acrylonitrile exceeded the standard value of 1.00 × 10^−6^ for all the vehicles for women users on weekdays: A, 2.22 × 10^−6^; B, 1.44 × 10^−6^; C, 5.06 × 10^−6^; D, 2.97 × 10^−6^; E, 2.87 × 10^−6^; F, 1.52 × 10^−6^; G, 1.50 × 10^−6^; and H, 2.23 × 10^−6^. The value for 1,2-dichloroethane was 1.98 × 10^−6^ for vehicle D, exceeding its standard limit. Formaldehyde exceeded its standard value of 1.00 × 10^−6^ for vehicles B, C, D, F, G, and H with values of 1.44 × 10^−6^, 1.02 × 10^−6^, 1.04 × 10^−6^, 1.03 × 10^−6^, 1.09 × 10^−6^, and 1.28 × 10^−6^, respectively.

For the category men and weekend, acrylonitrile exceeded the standard of 1.00 × 10^−6^ for vehicles C, D, and E with values of 2.14 × 10^−6^, 1.26 × 10^−6^, and 1.21 × 10^−6^, respectively, but 1,2-dichloroethane, benzene, acetaldehyde, and formaldehyde did not exceed their standard limit in any of the vehicles.

In the case of men and weekdays, acrylonitrile exceeded the standard of 1.00 × 10^−6^ for all the vehicles: A, 2.91 × 10^−6^; B, 1.88 × 10^−6^; C, 6.62 × 10^−6^; D, 3.88 × 10^−6^; E, 3.75 × 10^−6^; F, 1.99 × 10^−6^; G, 1.96 × 10^−6^; and H, 2.92 × 10^−6^. The concentration of 1,2-dichloroethane in vehicle D was 2.60 × 10^−6^, exceeding its standard limit. Acetaldehyde also exceeded its standard limit in vehicles E and F with the values 1.30 × 10^−6^ and 1.13 × 10^−6^, respectively. Formaldehyde exceeded its standard limit in all vehicles, except for vehicle A.

For men and weekend, acrylonitrile exceeded its standard of 1.00 × 10^−6^ for vehicles A, C, D, E, F, G, and H with values of 1.48 × 10^−6^, 3.36 × 10^−6^, 1.97 × 10^−6^, 1.90 × 10^−6^, 1.01 × 10^−6^, and 1.48 × 10^−6^, respectively. That of 1,2-dichloroethane exceeded the criterion with a value of 1.32 × 10^−6^ for vehicle D. Benzene, acetaldehyde, and formaldehyde did not exceed their criteria in any of the vehicles.

#### 3.3.2. Non-Carcinogens

HQ_chronic_ was calculated for the non-carcinogenic target compounds in the health risk assessment: acrylonitrile, 1,1-dichloroethylene, methylene chloride, allyl chloride, chloroform, 1,2-dichloroethane, benzene, carbon tetrachloride, 1,2-dichloropropane, trichloroethylene, toluene, 1,2-dibromoethane, tetrachloroethylene, ethyl benzene, m-xylene, p-xylene, o-xylene, styrene, 1,3,5-trimethylbenzene, 1,2,4-trimethylbenzene, methyl ethyl ketone, methyl isobutyl ketone, hexane, acetaldehyde, acrolein, propionaldehyde, ammonia, and hydrogen sulfide. In this instance, HQ_chronic_ was calculated only for 17 substances that had non-carcinogenic toxicity values and detectable concentrations (acrylonitrile, benzene, toluene, ethyl benzene, m-xylene, p-xylene, o-xylene, styrene, 1,3,5-trimethylbenzene, 1,2,4-trimethylbenzene, methyl ethyl ketone, methyl isobutyl ketone, hexane, acetaldehyde, propionaldehyde, ammonia, and hydrogen sulfide).

**Figure 4:**
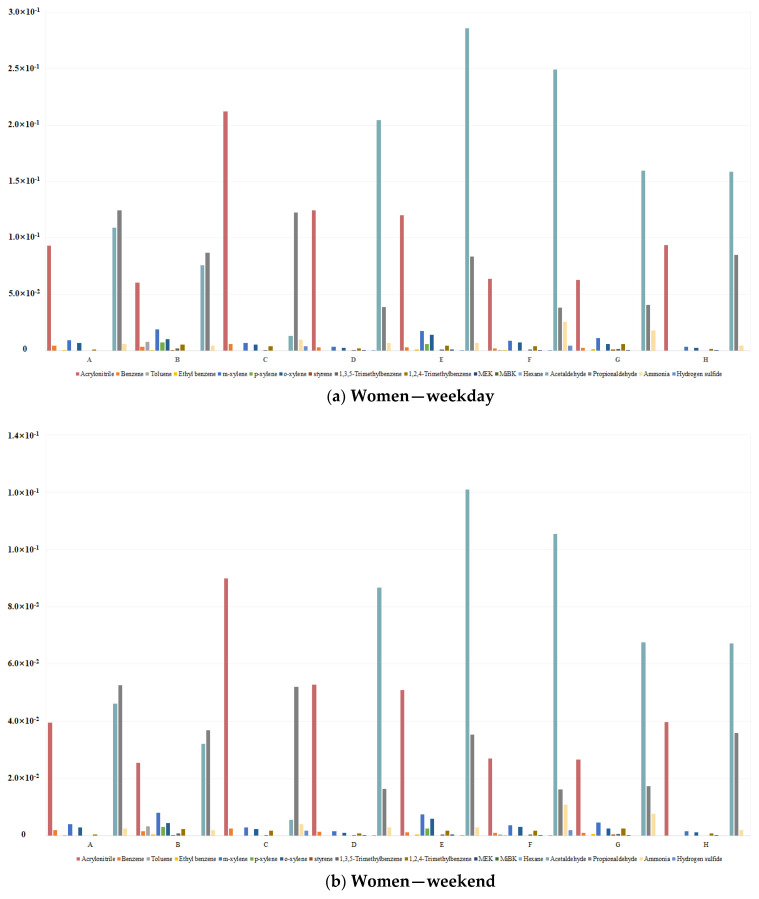

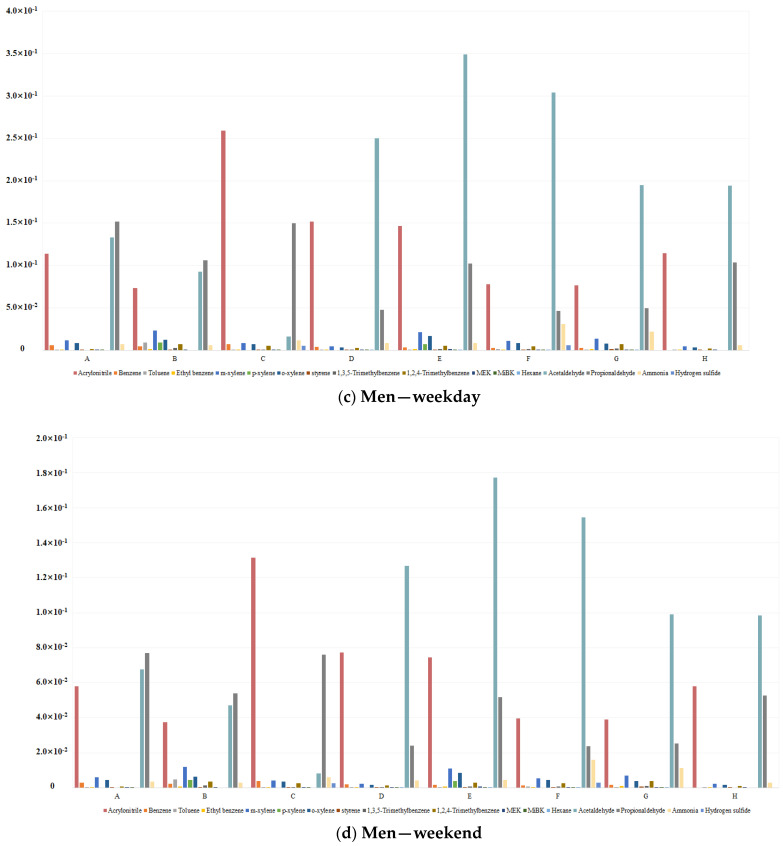
Chronic health risk assessment results for non-carcinogens.

In the case of chronic non-carcinogenic health risk assessment, the risk was calculated by distinguishing sex (women and men) and day of the week (weekdays and weekends) according to the driving characteristics. The results are listed in Table S6 and Figure 4.

No pollutants exceeded the standard value of 1 across any demographic or temporal category. The total risk values, combining weekday and weekend exposure for both women and men, remained below the criteria, suggesting minimal non-carcinogenic health risks. 

## 4. Discussion

In this study, the pollutant exposure potential for automobile users was calculated by measuring pollutant concentrations inside newly manufactured automobiles. This is the first study in Korea to conduct health risk assessment of acute and chronic pollutant exposure according to the environmental characteristics of automobiles.

Although 81 pollutants were investigated, most previous studies on automotive VOCs have focused primarily on benzene, toluene, ethyl benzene, and xylene (BTEX). Comparing BTEX concentrations with previous studies revealed that our measurements were lower than those reported by Yoshida and Matsunaga [27] and Tong et al. [28] but higher than that reported by Lau et al. [29], Jo and Yu [30], and Tokumura et al. [31]. In general, most of the chemicals detected in automotive cabins are reported to occur from existing materials, and new vehicles are known to exhibit higher concentrations of organic compounds than older vehicles. In a previous study, multiple linear regression revealed that the R^2^ value was 0.6 or higher for the concentrations of most compounds inside automobiles and three factors (internal temperature, internal humidity, and the number of days after release). Therefore, the internal temperature and the number of days after release are considered the main factors to affect indoor pollutant concentrations [27]. Accordingly, the results of Yoshida and Matsunaga [27] and those of the present study reporting high VOC concentrations inside new cars can be attributed to the high concentration of organic compounds inside new cars. Tokumura et al. [31] also investigated the IAQ of new cars, but the temperature during sampling was in the range of 14.3–23.0 °C. Since a high-temperature environment may increase the concentrations of VOCs by increasing the organic compound release rate of materials [26,32,33,34,35], this explains why Tokumura et al. [31] obtained lower concentrations of VOCs compared with the present study even though both studies measured VOCs in new cars.

The health risk assessment revealed that ECR_chronic_ values for acrylonitrile and formaldehyde frequently exceeded the standard limit of 1.00 × 10^−6^, indicating potential adverse health effects. Liang et al. [36] found formaldehyde as a “possible risk (1.00 × 10^−6^–1.00 × 10^−5^)” inside most vehicles, whereas benzene and acetaldehyde mostly exhibited “acceptable risk (less than 1.00 × 10^−6^)”, which is consistent with the results of the present study. Xu et al. [37] conducted a health risk assessment on parked vehicles exposed to sunlight to evaluate the worst-case scenario under which large amounts of pollutants are emitted in automotive cabins and found that formaldehyde exhibited the highest risk, with a value of 4.92 × 10^−5^–6.01 × 10^−7^. In addition, the health risk calculation results by age showed that the risk for middle-aged adults (18 to 60 years old) and children (less than 6 years) is higher than that for younger (6 to 18 years) and older adults (60 years and over). This appears to be because middle-aged adults (18 to 60 years old) spend considerable time in vehicles due to work, and children are more sensitive to pollutants. In the present study, the carcinogenic risk of formaldehyde was found to be 1.88 × 10^−6^–2.94 × 10^−7^, which was lower than that reported by Xu et al. [37]. This may be because Xu et al. [37] conducted the health risk assessment under the worst-case scenario of sunlight exposure to parked cars.

The main means of transportation for Koreans are passenger cars (52%), buses (15.4%), subways (10.3%), and bicycles [38]. Xu et al. [37] measured and analyzed the concentration of carbonyl compounds in 20 cars and 11 public transportation vehicles (six subways and five buses) and found a significant difference in pollutant detection rate between cars and public transportation vehicles. In general, pollutant concentrations are much higher in automobiles than in public transport vehicles [37,39,40] meaning that people can be exposed to more pollutants in automobiles than in public transportation. Therefore, health effects can differ for people in automobiles and public transportation vehicles for the same amount of time. From the perspective of driver health protection, it is necessary to conduct exposure and health risk assessment for various pollutants in automobiles.

This study has some limitations. Since pollutants in automotive cabins were measured only once for each vehicle in this study, there can be uncertainties about the measurements. Additionally, the emitted pollutants may vary depending on the materials of the different vehicle parts [36], considering that the materials and processes used in the automobile manufacturing process can be different. VOCs inside the vehicles are expected to vary significantly depending on the vehicle, but the composition of VOCs according to car parts was not analyzed because this study aimed to conduct health risk assessment due to acute and chronic exposure that reflects the exposure characteristics of automobile users. Future studies should measure VOCs according to the material and manufacturing process of vehicle parts. This can reduce the health effects on automobile users by identifying sources for each pollutant. In addition, among the measured pollutants inside vehicles in this study, toluene exhibited the highest concentration (203.5 ± 379.3 μg/m^3^), whereas the concentration of acrylonitrile (6.4 ± 2.9 μg/m^3^) was considerably low. However, the health risk assessment results indicated that toluene had no possibility of causing health risks, while acrylonitrile may lead to adverse health effects. These results indicate the need for management that considers not only the concentrations of pollutants inside automobiles but also the health effects of these pollutants on automobile users.

The results of this study revealed that the studied pollutants have no acute health effects or chronic non-carcinogenic health effects on automobile users; however, some of these pollutants may cause chronic carcinogenic health effects. This study also confirmed that there is a difference in health risk between women and men despite both sexes being exposed to the same concentration. This is because the exposure coefficients of women and men for automobile driving are different. However, in this study, health risk assessment of pollutants was conducted by using the exposure coefficients derived from a survey of automobile users; thus, a customized exposure amount was calculated for automobile users. In addition, previous studies have demonstrated that the concentrations of pollutants inside automobiles decrease over time. However, in this study, we measured the IAQ inside new cars and conducted exposure and health risk assessment when the pollutants were present in high concentrations. Nevertheless, our results are valuable in that a conservative assessment was conducted from the perspective of health protection.

## 5. Conclusions

This study analyzed pollutant concentrations in newly manufactured automobiles and evaluated health effects of acute and chronic exposure using exposure factors that reflect automobile user characteristics.

Among 81 target pollutants, 52 were detected inside the eight vehicles, and 30 had values below detection limit. Toluene exhibited the highest concentration (203.5 ± 379.3 μg/m^3^). In the acute health risk assessment, none of the pollutants exceeded their standard limits in any of the vehicles, indicating that there is no possibility of causing health effects. In the chronic carcinogenic health risk assessment, acrylonitrile exceeded its standard value of 1.00 × 10^−6^ in all the vehicles, in the case of weekdays, for women and men. Formaldehyde exhibited the second-highest risk as it exceeded its standard for six vehicles in the case of women and weekdays and for seven vehicles in the case of men and weekdays. In the chronic non-carcinogenic health risk assessment, no pollutant exceeded its standard value for any of the vehicles in the cases of women and weekdays, women and weekends, men and weekdays, and men and weekends, indicating that there is no possibility of developing health effects. The HI calculation result, which is the sum of the hazard quotients (HQs) of individual pollutants, also did not exceed the criterion of 1.

Therefore, the estimation of the health effects of lifetime exposure to pollutants inside cars should receive more attention. In the future, more detailed studies need to be conducted on the pollutants generated by the materials and manufacturing processes of vehicle components. This can reduce the health effects on automobile users by identifying the sources of such pollutants. Additionally, it is believed that a health risk assessment study that takes into account the decrease in pollutant concentration over time inside the automobile is necessary.

## Figures and Tables

**Table 1:** Acute health risk assessment results for pollutants by vehicle.

	A	B	C	D	E	F	G	H
Benzene	1.65 × 10^−1^	1.28 × 10^−1^	2.06 × 10^−1^	1.10 × 10^−1^	1.02 × 10^−1^	8.42 × 10^−2^	8.61 × 10^−2^	-
Toluene	7.69 × 10^−3^	2.40 × 10^−1^	1.09 × 10^−2^	8.02 × 10^−3^	1.29 × 10^−2^	3.08 × 10^−2^	9.37 × 10^−3^	5.47 × 10^−3^
m-Xylene	1.36 × 10^−3^	2.70 × 10^−3^	9.83 × 10^−4^	5.40 × 10^−4^	2.48 × 10^−3^	1.26 × 10^−3^	1.57 × 10^−3^	5.24 × 10^−4^
p-Xylene	-	1.03 × 10^−3^	-	-	8.78 × 10^−4^	-	-	-
o-Xylene	9.94 × 10^−4^	1.46 × 10^−3^	8.19 × 10^−4^	3.74 × 10^−4^	1.97 × 10^−3^	1.02 × 10^−3^	8.84 × 10^−4^	4.13 × 10^−4^
Styrene	7.27 × 10^−4^	1.06 × 10^−3^	3.90 × 10^−4^	2.91 × 10^−4^	2.75 × 10^−4^	2.27 × 10^−4^	1.68 × 10^−3^	3.71 × 10^−4^
1,3,5-Trimethylbenzene	-	5.70 × 10^−3^	2.16 × 10^−3^	1.57 × 10^−3^	3.47 × 10^−3^	3.09 × 10^−3^	4.19 × 10^−3^	-
1,2,4-Trimethylbenzene	2.82 × 10^−3^	1.48 × 10^−2^	1.09 × 10^−2^	5.26 × 10^−3^	1.17 × 10^−2^	1.07 × 10^−2^	1.60 × 10^−2^	4.92 × 10^−3^
MEK	2.58 × 10^−3^	1.25 × 10^−3^	2.14 × 10^−3^	8.44 × 10^−3^	1.32 × 10^−2^	8.25 × 10^−3^	6.33 × 10^−3^	7.81 × 10^−3^
Formaldehyde	2.03 × 10^−1^	4.20 × 10^−1^	3.00 × 10^−1^	3.04 × 10^−1^	2.82 × 10^−1^	3.01 × 10^−1^	3.19 × 10^−1^	3.73 × 10^−1^
Acetaldehyde	6.48 × 10^−2^	4.52 × 10^−2^	8.00 × 10^−3^	1.21 × 10^−1^	1.70 × 10^−1^	1.48 × 10^−1^	9.48 × 10^−2^	9.42 × 10^−2^
Ammonia	2.85 × 10^−2^	2.31 × 10^−2^	4.79 × 10^−2^	3.33 × 10^−2^	3.41 × 10^−2^	1.24 × 10^−1^	8.83 × 10^−2^	2.27 × 10^−2^
Hydrogen sulfide	-	-	6.31 × 10^−3^	-	-	6.97 × 10^−3^	-	-

## Data Availability

Restrictions apply to the availability of these data. Data were obtained from MOLIT and are available with the permission of MOLIT.

## Supplementary Materials

The following supporting information can be downloaded at: https://www.mdpi.com/article/10.3390/toxics12120843/s1, Table S1: Quality assurance and control results; Table S2: Acute and chronic toxicity information for the target pollutants; Table S3: Exposure factors of this study; Table S4: Pollutant concentrations inside the vehicles; Table S5: Chronic health risk assessment results for carcinogens; Table S6: Chronic health risk assessment results for carcinogens.
