# Supplementary material for: Acute and Chronic Health Risk Assessment for Automobile Users Due to Inhalation Exposure to Volatile Organic Compounds and Carbonyl Compounds
Source: Toxics. 2024 Nov 24;12(12):843. doi: 10.3390/toxics12120843 (PMC11679100; doi:10.3390/toxics12120843)
Supplement: Supplementary file 1 [file toxics-12-00843-s001.zip › toxics-3248601-supplementary.pdf]

Table S1. Quality assurance and control results

Substance	Linearity	Detection limit (μg/m³)	Quantification limit (μg/m³)	Analysis precision [RT]	Analysis precision [area]
Acrylonitrile	0.9986	3.4	11.0	0.002	2.7
1,1-Dichloroethylene	0.9995	8.3	26.4	0.001	3.5
Methylene chloride	0.9999	6.4	20.4	0.001	3.9
Allyl chloride	0.9949	1.8	5.8	0.001	3.3
1,1-Dichloroethane	1.0000	7.0	22.3	0.007	2.0
cis-1,2-Dichloroethylene	0.9982	7.9	25.1	0.007	2.1
Chloroform	0.9982	10.8	34.3	0.008	2.1
1,2-Dichloroethane	1.0000	11.8	37.6	0.007	1.9
1,1,1-Trichloroethane	0.9978	13.9	44.2	0.007	2.5
Benzene	0.9999	1.7	5.3	0.006	2.0
Carbon tetrachloride	0.9992	15.3	48.6	0.006	2.3
1,2-Dichloropropane	0.9997	3.8	12.0	0.005	2.0
Trichloroethylene	1.0000	7.4	23.5	0.004	1.9
cis-1,3-Dichloropropene	0.9993	4.1	13.0	0.003	2.5
trans-1,3-Dichloropropene	0.9993	5.1	16.2	0.003	2.9
1,1,2-Trichloroethane	0.9999	5.8	18.5	0.003	2.3
Toluene	0.9998	5.3	17.0	0.002	2.5
1,2-Dibromoethane	0.9997	6.5	20.7	0.002	2.8
Tetrachloroethylene	1.0000	7.5	23.8	0.002	2.2
Chlorobenzene	0.9999	5.1	16.4	0.002	2.6
Ethyl benzene	0.9999	5.3	16.9	0.003	2.6
m-Xylene	1.0000	9.9	31.5	0.003	5.5
p-Xylene	0.9996	9.1	29.0	0.003	1.5
Styrene	0.9997	2.9	9.3	0.003	3.0
1,1,2,2-Tetrachloroethane	1.0000	7.7	24.4	0.003	2.6
o-Xylene	1.0000	5.8	18.3	0.003	2.7
4-Ethyltoluene	0.9995	2.4	7.6	0.002	3.3
1,3,5-Trimethylbenzene	0.9988	3.5	11.2	0.002	3.3
1,2,4-Trimethylbenzene	0.9987	2.5	8.0	0.001	3.2
1,3-Dichlorobenzene	0.9995	4.7	15.0	0.001	3.9
1,4-Dichlorobenzene	0.9992	3.2	10.2	0.001	3.7
1,2-Dichlorobenzene	0.9993	4.8	15.4	0.001	3.5

1,2,4-Trichlorobenzene	1.0000	9.7	30.8	0.003	5.2
Hexachloro-1,3-butadiene	1.0000	15.6	49.8	0.003	3.4
Methyl ethyl ketone	0.998	5.9	18.7	0.007	2.3
Iso-butanol	0.9995	6.7	21.5	0.007	1.9
Methyl isobutyl ketone	1.0000	5.9	18.7	0.004	1.9
n-Butyl acetate	1.0000	6.2	19.9	0.003	2.2
2-Propanol	0.9835	4.6	14.5	0.002	2.8
1-Propanol	0.9999	4.0	12.8	0.004	1.9
Ethyl acetate	0.9989	4.4	14.0	0.006	2.6
Hexane	0.9922	8.9	28.5	0.006	5.9
n-Butanol	0.9974	3.6	11.6	0.005	3.9
Bromodichloromethane	0.9949	3.8	12.1	0.005	4.3
n-Heptane	0.9994	3.5	11.1	0.004	2.1
Dibromochloromethane	0.9979	3.4	10.9	0.003	3.7
n-Octane	0.9993	3.8	12.1	0.004	1.2
n-Nonane	0.9999	4.0	12.7	0.003	1.7
(1S)-(−)-Alpha-pinene, synthetic	0.9976	3.1	10.0	0.003	1.3
3-Ethyltoluene	0.9991	3.9	12.4	0.002	1.5
2-Ethyltoluene	0.9996	3.5	11.2	0.002	0.9
(−)-Beta-pinene	0.9982	3.2	10.1	0.002	1.4
n-Decane	0.9998	3.7	11.8	0.001	1.2
1,2,3-Trimethylbenzene	0.9997	3.4	10.8	0.002	1.4
(R)-(+)-Limonene	1.0000	3.5	11.2	0.001	0.9
Nonanal	0.9999	4.7	14.9	0.001	1.1
n-Undecane	0.9997	4.1	13.4	0.001	1.1
1,2,4,5-Tetramethylbenzene	0.9996	3.8	12.0	0.001	2.1
Decanal, synthetic	0.9993	5.4	17.3	0.001	0.7
n-Dodecane	0.9951	3.8	12.2	0.001	1.2
n-Tridecane	0.9979	3.8	12.0	0.002	1.0
n-Tetradecane	0.9986	3.9	12.4	0.003	1.3
n-Pentadecane	0.9998	5.4	17.2	0.004	0.7
n-Hexadecane	1.0000	4.8	15.3	0.004	0.4
Formaldehyde	1.0000	0.2	0.5	0.001	0.3
Acetaldehyde	1.0000	0.2	0.6	0.001	0.2
Acrolein	1.0000	0.3	0.9	0.002	0.3
Acetone	1.0000	0.4	1.2	0.002	0.3

Propionaldehyde	1.0000	0.4	1.3	0.003	0.6
Crotonaldehyde	1.0000	0.7	2.3	0.002	0.1
Methacrolein	1.0000	0.4	1.4	0.002	0.5
Butyraldehyde	1.0000	0.6	2.1	0.002	0.4
Benzaldehyde	1.0000	0.8	2.5	0.002	0.9
Iso-valeraldehyde	0.9998	0.2	0.6	0.001	0.2
Valeraldehyde	1.0000	0.4	1.2	0.002	0.5
m-Tolualdehyde	1.0000	0.9	2.8	0.001	0.2
Hexaldehyde	1.0000	0.4	0.1	0.002	0.5
Trimethylamine	0.9986	0.4	0.1	0.001	0.5
Hydrogen sulfide	0.9957	0.2	0.7	0.000	2.6
Methyl mercaptan	0.9986	0.3	1.1	0.010	1.1
Dimethyl sulfide	0.9964	0.5	1.6	0.012	1.6
Dimethyl disulfide	0.9951	0.7	2.1	0.006	0.3
Ammonia	0.9999	0.6	1.8	-	0.1

Table S2. Acute and chronic toxicity information for the target pollutants

Pollutants	Inhalation Reference Value		
	Acute* ($\mu g/m^3$)	Chronic	
		Cancer** ($(\mu g/m^3)^{-1}$)	Non-cancer*** (mg/m^3)
Acrylonitrile	-	6.8×10^{-5}	2.0×10^{-3}
1,1-Dichloroethylene	-	-	2.0×10^{-1}
Methylene chloride	14,000	1.0×10^{-8}	6.0×10^{-1}
Allyl chloride	-	-	1.0×10^{-3}
Chloroform	150	2.3×10^{-5}	1.0×10^{-2}
1,2-Dichloroethane	-	2.6×10^{-5}	9.0×10^{-3}
1,1,1-Trichloroethane	-	-	-
Benzene	27	7.8×10^{-6}	3.0×10^{-2}
Carbon Tetrachloride	1,900	6.0×10^{-6}	1.0×10^{-1}
1,2-Dichloropropane	-	-	4.0×10^{-3}
Trichloroethylene	-	4.1×10^{-6}	2.0×10^{-3}
cis-1,3-Dichloropropene	-	-	-
trans-1,3-Dichloropropene	-	-	-
1,1,2-Trichloroethane	-	1.6×10^{-5}	-
Toluene	5,000	-	5.0
1,2-Dibromoethane	-	3.0×10^{-4}	9.0×10^{-3}
Tetrachloroethylene	20,000	2.6×10^{-7}	4.0×10^{-2}
Ethyl benzene	-	-	1.0
m-Xylene	22,000	-	1.0×10^{-1}
p-Xylene	22,000	-	1.0×10^{-1}
o-Xylene	22,000	-	1.0×10^{-1}
Styrene	21,000	-	1.0
1,3,5-Trimethylbenzene	2,400	-	2.0×10^{-1}
1,2,4-Trimethylbenzene	2,400	-	2.0×10^{-1}
Hexachloro-1,3-butadiene	-	2.2×10^{-5}	-
Methyl ethyl ketone	13,000	-	5.0
Iso-butanol	-	-	-

Methyl isobutyl ketone	-	-	3.0
Hexane	-	-	7.0×10^{-1}
Formaldehyde	55	1.3×10^{-5}	-
Acetaldehyde	470	2.2×10^{-6}	9.0×10^{-3}
Acrolein	2.5	-	2.0×10^{-5}
Propionaldehyde	-	-	8.0×10^{-3}
Ammonia	3,200	-	5.0×10^{-1}
Hydrogen sulfide	42	-	2.0×10^{-3}

*OEHHA (Office of Environmental Health Hazard Assessment of California EPA)'s acute reference exposure level (REL)
**US EPA IRIS (Integrated Risk Information System)'s inhalation unit risk (IUR)
***US EPA IRIS (Integrated Risk Information System)'s reference concentration (RfC)

Table S3. Exposure factors of this study

Parameter	Definition	Sex	Value		Reference
			Weekday	Weekend	
IR	Inhalation rate (m^3/day)	Female	13.03		MoE (2019)
		Male	16.21		
EF	Exposure frequency (day/year)	Female	197.6	67.6	This study
		Male	202.8	78.0	
ED	Exposure duration (year)	Female	15		KADRA (2015)
		Male			
ET	Exposure time	Female	0.0754	0.0933	This study
		Male	0.0894	0.118	
BW	Body weight (kg)	Female	57.7		MoE (2019)
		Male	71.5		
AT	Average time (day)	Female	5,475		KADRA (2015)
		Male			
LT	Lifetime (day)	Female	31,244		KOSTAT (2021)
		Male	29,164		

Table S4. Pollutant concentrations inside the vehicles

Pollutants	Detection Frequency (%)	Concentration Distribution (μg/m³)		
		Mean ± S.D.	Min	Max
Acrylonitrile	100	6.4 ± 2.9	3.7	13.2
1,1-Dichloroethylene	0	N.D.	N.D.	N.D.
Methylene chloride	0	N.D.	N.D.	N.D.
Allyl chloride	0	N.D.	N.D.	N.D.
1,1-Dichloroethane	0	N.D.	N.D.	N.D.
cis-1,2-Dichloroethylene	0	N.D.	N.D.	N.D.
Chloroform	0	N.D.	N.D.	N.D.
1,2-Dichloroethane	12.5	13.5 ± 0	13.5	N.D.
1,1,1-Trichloroethane	0	N.D.	N.D.	N.D.
Benzene	87.5	3.4 ± 1.1	2.3	5.6
Carbon tetrachloride	0	N.D.	N.D.	N.D.
1,2-Dichloropropane	0	N.D.	N.D.	N.D.
Trichloroethylene	0	N.D.	N.D.	N.D.
cis-1,3-Dichloropropene	0	N.D.	N.D.	N.D.
trans-1,3-Dichloropropene	0	N.D.	N.D.	N.D.
1,1,2-Trichloroethane	0	N.D.	N.D.	N.D.
Toluene	100	203.5 ± 379.3	27.3	1202.3
1,2-Dibromoethane	0	N.D.	N.D.	N.D.
Tetrachloroethylene	0	N.D.	N.D.	N.D.
Chlorobenzene	0	N.D.	N.D.	N.D.
Ethyl benzene	100	24.6 ± 12.8	11.2	46.4
m-Xylene	100	31.4 ± 16.7	11.5	59.5
p-Xylene	25.0	21.0 ± 1.7	19.3	22.7
Styrene	100	13.2 ± 10.1	4.8	35.4
1,1,2,2-Tetrachloroethane	0	N.D.	N.D.	N.D.
o-Xylene	100	21.9 ± 10.9	8.2	43.4
4-Ethyltoluene	75.0	5.1 ± 1.4	3.0	6.8
1,3,5-Trimethylbenzene	75.0	8.1 ± 3.2	3.8	13.7
1,2,4-Trimethylbenzene	100	23.1 ± 10.8	6.8	38.4
1,3-Dichlorobenzene	0	N.D.	N.D.	N.D.
1,4-Dichlorobenzene	0	N.D.	N.D.	N.D.
1,2-Dichlorobenzene	12.5	7.3 ± 0.0	7.3	N.D.
1,2,4-Trichlorobenzene	0	N.D.	N.D.	N.D.
Hexachloro-1,3-butadiene	0	N.D.	N.D.	N.D.
MEK	100	81.3 ± 49.4	16.2	172.0
i-BuOH	50.0	19.8 ± 10.8	7.2	36.9
MIBK	75.0	11.6 ± 3.8	6.5	17.6

n-Butyl acetate	100	44.5 ± 41.9	7.2	138.3
2-Propanol	100	11.6 ± 4.4	5.9	19.0
1-Propanol	0	N.D.	N.D.	N.D.
Ethyl acetate	100	30.9 ± 26.7	6.9	82.4
Hexane	57.1	16.6 ± 4.2	9.5	20.2
n-Butanol	100	107.3 ± 45.0	45.4	198.7
Bromodichloromethane	0	N.D.	N.D.	N.D.
n-Heptane	57.1	5.1 ± 1.3	3.8	6.5
Diboromochloromethane	0	N.D.	N.D.	N.D.
n-Octane	100	19.9 ± 20.0	5.7	72.1
n-Nonane	42.9	24.8 ± 2.0	4.7	55.5
(1S)-(−)-Alpha-pinene, synthetic	42.9	6.0 ± 3.0	3.6	10.1
3-Ethyltoluene	71.4	8.4 ± 2.0	6.1	11.6
2-Ethyltoluene	57.1	4.3 ± 0.4	3.6	4.8
(−)-Beta-pinene	14.3	3.5 ± 0.0	3.5	N.D.
n-Decane	100	181.4 ± 251.3	37.6	792.0
1,2,3-Trimethylbenzene	71.4	6.3 ± 1.1	5.2	7.9
(R)-(+)-Limonene	57.1	5.1 ± 0.9	3.9	6.4
Nonanal	100	38.3 ± 25.4	8.2	87.7
n-Undecane	100	134.5 ± 137.1	10.1	353.2
1,2,4,5-Tetramethylbenzene	14.3	5.1 ± 0.0	5.1	N.D.
Decanal, synthetic	14.3	6.0 ± 0.0	6.0	N.D.
n-Dodecane	100	63.1 ± 70.3	9.7	212.6
n-Tridecane	71.4	6.2 ± 1.2	4.9	8.4
n-Tetradecane	57.1	5.8 ± 1.2	4.7	7.8
n-Pentadecane	0	N.D.	N.D.	N.D.
n-Hexadecane	0	N.D.	N.D.	N.D.
Formaldehyde	100	17.2 ± 3.3	11.2	23.1
Acetaldehyde	100	43.8 ± 23.5	3.8	79.7
Acrolein	0	N.D.	N.D.	N.D.
Acetone	100	74.9 ± 36.6	21.0	133.0
Propionaldehyde	100	19.2 ± 8.2	9.5	30.8
Crotonaldehyde	0	N.D.	N.D.	N.D.
Methacrolein	0	N.D.	N.D.	N.D.
Butyraldehyde	100	13.3 ± 3.3	10.3	21.0
Benzaldehyde	87.5	6.8 ± 3.8	2.1	12.2
Iso-valeraldehyde	12.5	9.5 ± 0.0	9.5	N.D.
Valeraldehyde	75.0	4.4 ± 1.5	1.8	6.5
m-Tolualdehyde	12.5	4.7 ± 0.0	4.7	N.D.
Hexaldehyde	87.5	16.3 ± 9.6	5.3	29.5
Trimethylamine	100	1.0 ± 1.1	0.1	3.1

Ammonia	100	160.8 ± 109.8	72.7	397.0
Hydrogen sulfide	25.0	0.3 ± 0.0	0.3	N.D.
Methyl mercaptan	0	N.D.	N.D.	N.D.
Dimethyl sulfide	0	N.D.	N.D.	N.D.
Dimethyl disulfide	0	N.D.	N.D.	N.D.
TVOC	100	8813.6 ± 8868.7	4034.0	31962.1

Table S5. Chronic health risk assessment results for carcinogens

Pollutant	Type	$ECR_{chronic}$					
		Women			Men		
		Weekday	Weekend	Total	Weekday	Weekend	Total
Acrylonitrile	A	2.22E-06	9.42E-07	3.17E-06	2.91E-06	1.48E-06	4.39E-06
	B	1.44E-06	6.08E-07	2.04E-06	1.88E-06	9.54E-07	2.83E-06
	C	5.06E-06	2.14E-06	7.20E-06	6.62E-06	3.36E-06	9.98E-06
	D	2.97E-06	1.26E-06	4.22E-06	3.88E-06	1.97E-06	5.85E-06
	E	2.87E-06	1.21E-06	4.08E-06	3.75E-06	1.90E-06	5.66E-06
	F	1.52E-06	6.44E-07	2.16E-06	1.99E-06	1.01E-06	3.00E-06
	G	1.50E-06	6.35E-07	2.14E-06	1.96E-06	9.97E-07	2.96E-06
	H	2.23E-06	9.46E-07	3.18E-06	2.92E-06	1.48E-06	4.41E-06
1,2-Dichloroethane	A	-	-	-	-	-	-
	B	-	-	-	-	-	-
	C	-	-	-	-	-	-
	D	1.98E-06	8.40E-07	2.82E-06	2.60E-06	1.32E-06	3.91E-06
	E	-	-	-	-	-	-
	F	-	-	-	-	-	-
	G	-	-	-	-	-	-
	H	-	-	-	-	-	-
Benzene	A	1.97E-07	8.33E-08	2.80E-07	2.58E-07	1.31E-07	3.88E-07
	B	1.53E-07	6.46E-08	2.17E-07	2.00E-07	1.01E-07	3.01E-07
	C	2.46E-07	1.04E-07	3.50E-07	3.22E-07	1.63E-07	4.85E-07
	D	1.31E-07	5.55E-08	1.87E-07	1.72E-07	8.71E-08	2.59E-07
	E	1.21E-07	5.14E-08	1.73E-07	1.59E-07	8.07E-08	2.40E-07
	F	1.00E-07	4.25E-08	1.43E-07	1.31E-07	6.66E-08	1.98E-07
	G	1.03E-07	4.34E-08	1.46E-07	1.34E-07	6.81E-08	2.02E-07
	H	-	-	-	-	-	-
Acetaldehyde	A	3.79E-07	1.60E-07	5.39E-07	4.96E-07	2.52E-07	7.48E-07
	B	2.64E-07	1.12E-07	3.76E-07	3.46E-07	1.75E-07	5.21E-07
	C	4.68E-08	1.98E-08	6.66E-08	6.12E-08	3.11E-08	9.23E-08
	D	7.10E-07	3.01E-07	1.01E-06	9.29E-07	4.72E-07	1.40E-06
	E	9.91E-07	4.20E-07	1.41E-06	1.30E-06	6.59E-07	1.96E-06
	F	8.65E-07	3.66E-07	1.23E-06	1.13E-06	5.74E-07	1.71E-06

	G	5.54E-07	2.35E-07	7.89E-07	7.25E-07	3.68E-07	1.09E-06
	H	5.51E-07	2.33E-07	7.84E-07	7.21E-07	3.66E-07	1.09E-06
Formaldehyde	A	6.95E-07	2.94E-07	9.89E-07	9.10E-07	4.62E-07	1.37E-06
	B	1.44E-06	6.08E-07	2.05E-06	1.88E-06	9.55E-07	2.84E-06
	C	1.02E-06	4.34E-07	1.46E-06	1.34E-06	6.81E-07	2.02E-06
	D	1.04E-06	4.40E-07	1.48E-06	1.36E-06	6.90E-07	2.05E-06
	E	9.65E-07	4.09E-07	1.37E-06	1.26E-06	6.41E-07	1.90E-06
	F	1.03E-06	4.36E-07	1.47E-06	1.35E-06	6.85E-07	2.03E-06
	G	1.09E-06	4.62E-07	1.55E-06	1.43E-06	7.25E-07	2.15E-06
	H	1.28E-06	5.40E-07	1.82E-06	1.67E-06	8.47E-07	2.52E-06

Table S6. Chronic health risk assessment results for carcinogens

Pollutant	Type	$HQ_{chronic}$					
		Women			Men		
		Weekday	Weekend	Total	Weekday	Weekend	Total
Acrylonitrile	A	9.33E-02	3.95E-02	1.33E-01	1.14E-01	5.79E-02	1.72E-01
	B	6.03E-02	2.55E-02	8.58E-02	7.36E-02	3.74E-02	1.11E-01
	C	2.12E-01	8.98E-02	3.02E-01	2.59E-01	1.32E-01	3.91E-01
	D	1.24E-01	5.27E-02	1.77E-01	1.52E-01	7.72E-02	2.29E-01
	E	1.20E-01	5.09E-02	1.71E-01	1.47E-01	3.96E-02	2.22E-01
	F	6.38E-02	2.70E-02	9.08E-02	7.80E-02	8.96E-02	1.18E-01
	G	6.30E-02	2.67E-02	8.96E-02	7.69E-02	3.90E-02	1.16E-01
	H	9.37E-02	3.97E-02	1.33E-01	1.15E-01	5.81E-02	1.73E-01
Benzene	A	4.80E-03	2.03E-03	6.83E-03	5.86E-03	2.98E-03	8.84E-03
	B	3.72E-03	1.57E-03	5.30E-03	4.45E-03	2.31E-03	6.85E-03
	C	5.99E-03	2.54E-03	8.53E-03	7.32E-03	3.72E-03	1.10E-02
	D	3.20E-03	1.35E-03	4.55E-03	3.91E-03	1.98E-03	5.89E-03
	E	2.96E-03	1.24E-03	4.21E-03	3.62E-03	1.84E-03	5.45E-03
	F	2.45E-03	1.04E-03	3.48E-03	2.99E-03	1.52E-03	4.51E-03
	G	2.50E-03	1.06E-03	3.53E-03	3.05E-03	1.55E-03	4.60E-03
	H	-	-	-	-	-	-
Toluene	A	2.48E-04	1.05E-04	3.53E-04	3.03E-04	1.54E-04	4.57E-04
	B	7.76E-03	3.28E-03	1.10E-02	9.48E-03	4.81E-03	1.43E-02
	C	3.51E-04	1.49E-04	5.00E-04	4.92E-04	2.18E-04	6.47E-04
	D	2.59E-04	1.10E-04	3.68E-04	3.16E-04	1.61E-04	4.77E-04
	E	4.17E-04	1.77E-04	5.94E-04	5.10E-04	2.59E-04	7.68E-04
	F	9.95E-04	4.21E-04	1.42E-03	1.22E-03	6.17E-04	1.83E-03
	G	3.02E-04	1.28E-04	4.30E-04	3.70E-04	1.88E-04	5.57E-04
	H	1.76E-04	7.47E-05	2.51E-04	2.15E-04	1.09E-04	3.25E-04
Ethyl benzene	A	6.28E-04	2.66E-04	8.93E-04	7.67E-04	3.89E-04	1.16E-03
	B	9.86E-04	4.17E-04	1.40E-03	1.20E-03	6.11E-04	1.82E-03
	C	3.61E-04	1.53E-04	5.13E-04	4.41E-04	2.21E-04	6.64E-04
	D	3.99E-04	1.69E-04	5.68E-04	4.87E-04	2.47E-04	7.35E-04
	E	1.37E-03	5.78E-04	1.94E-03	1.67E-03	8.47E-04	2.52E-03
	F	6.83E-04	2.89E-04	9.76E-04	8.35E-04	4.24E-04	1.26E-03

	G	1.50E-03	6.33E-04	2.13E-03	1.36E-02	9.28E-04	2.76E-03
	H	4.42E-04	1.87E-04	6.29E-04	4.54E-03	2.74E-04	8.14E-04
m-Xylene	A	9.62E-03	4.07E-03	1.37E-02	1.18E-02	5.97E-03	1.77E-02
	B	1.92E-03	8.12E-03	2.73E-02	2.34E-02	1.19E-02	3.53E-02
	C	6.98E-03	2.95E-03	9.93E-03	8.52E-03	4.33E-03	1.28E-02
	D	3.83E-03	1.62E-03	5.46E-03	4.68E-03	2.38E-03	7.06E-03
	E	1.76E-02	7.46E-03	2.51E-02	2.15E-02	1.09E-02	3.25E-02
	F	8.94E-03	3.78E-03	1.27E-02	1.09E-02	5.54E-03	1.65E-02
	G	1.11E-02	4.71E-03	1.58E-02	1.36E-02	6.90E-03	2.05E-02
	H	3.72E-03	1.57E-03	5.29E-03	4.54E-03	2.31E-03	6.85E-03
p-Xylene	A	-	-	-	-	-	-
	B	7.34E-03	3.11E-03	1.04E-02	8.96E-03	4.55E-03	1.35E-02
	C	-	-	-	-	-	-
	D	-	-	-	-	-	-
	E	6.24E-03	2.64E-03	8.87E-03	7.62E-03	3.87E-03	1.15E-02
	F	-	-	-	-	-	-
	G	-	-	-	-	-	-
	H	-	-	-	-	-	-
o-Xylene	A	7.05E-03	2.99E-03	1.00E-02	8.62E-03	4.38E-03	1.30E-02
	B	1.04E-02	4.40E-03	1.48E-02	1.27E-02	6.44E-03	1.91E-02
	C	5.81E-03	2.46E-03	8.27E-03	7.10E-03	3.61E-03	1.07E-02
	D	2.66E-03	1.12E-03	3.78E-03	3.25E-03	1.65E-03	4.89E-03
	E	1.40E-02	5.93E-03	1.99E-02	1.71E-02	8.69E-03	2.58E-02
	F	7.27E-03	3.08E-03	1.03E-02	8.88E-03	4.51E-03	1.34E-02
	G	6.27E-03	2.66E-03	8.93E-03	7.66E-03	3.98E-03	1.16E-02
	H	2.93E-03	1.24E-03	4.17E-03	3.58E-03	1.82E-03	5.39E-03
Styrene	A	4.93E-04	2.09E-04	7.01E-04	6.02E-04	3.06E-04	9.07E-04
	B	7.19E-04	3.05E-04	1.02E-03	8.79E-04	4.46E-04	1.33E-03
	C	2.65E-04	1.12E-04	3.77E-04	3.23E-04	1.64E-04	4.87E-04
	D	1.97E-04	8.36E-05	2.81E-04	2.41E-04	1.22E-04	3.64E-04
	E	1.86E-04	7.89E-05	2.65E-04	2.28E-04	1.16E-04	3.43E-04
	F	1.54E-04	6.51E-05	2.16E-04	1.88E-04	9.53E-05	2.83E-04
	G	1.14E-04	4.83E-04	1.62E-03	1.39E-03	7.08E-04	2.10E-03
	H	2.52E-04	1.06E-04	3.58E-04	3.07E-04	1.56E-04	4.63E-04

1,3,5-Trimethylbenzene	A	-	-	-	-	-	-
	B	2.21E-03	9.35E-04	3.14E-03	2.70E-03	1.37E-03	4.07E-03
	C	8.38E-04	3.55E-04	1.19E-03	1.02E-03	5.20E-04	1.54E-03
	D	6.09E-04	2.58E-04	8.67E-04	7.44E-04	3.78E-04	1.12E-03
	E	1.34E-03	5.69E-04	1.91E-03	1.64E-03	8.34E-04	2.48E-03
	F	1.19E-03	5.06E-04	1.70E-03	1.46E-03	7.41E-04	2.20E-03
	G	1.62E-03	6.86E-04	2.31E-03	1.98E-03	1.01E-03	2.99E-03
	H	-	-	-	-	-	-
1,2,4-Trimethylbenzene	A	1.09E-03	4.61E-04	1.55E-03	1.33E-03	6.76E-04	2.01E-03
	B	5.74E-03	2.43E-03	8.17E-03	7.01E-03	3.56E-03	1.06E-02
	C	4.22E-03	1.79E-03	6.00E-03	5.15E-03	2.62E-03	7.77E-03
	D	2.04E-03	8.62E-04	2.90E-03	2.49E-03	1.26E-03	3.75E-03
	E	4.51E-03	1.91E-03	6.42E-03	5.51E-03	2.80E-03	8.31E-03
	F	4.13E-03	1.75E-03	5.88E-03	5.05E-03	2.56E-03	7.61E-03
	G	6.20E-03	2.62E-03	8.82E-03	7.57E-03	3.84E-03	1.14E-02
	H	1.91E-03	8.07E-04	2.71E-03	2.33E-03	1.18E-03	3.51E-03
Methyl ethyl ketone	A	2.16E-04	9.16E-05	3.08E-04	2.64E-04	1.34E-04	3.99E-04
	B	1.04E-04	4.42E-05	1.49E-04	1.28E-04	6.48E-05	1.92E-04
	C	1.79E-04	7.60E-05	2.55E-04	2.19E-04	1.11E-04	3.31E-04
	D	7.08E-04	3.00E-04	1.01E-03	8.65E-04	4.39E-04	1.30E-03
	E	1.11E-03	4.70E-04	1.58E-03	1.36E-03	6.88E-04	2.04E-03
	F	6.92E-04	2.93E-04	9.85E-04	8.45E-04	4.29E-04	1.27E-03
	G	5.31E-04	2.25E-04	7.56E-04	6.49E-04	3.29E-04	9.78E-04
	H	6.55E-04	2.77E-04	9.32E-04	8.00E-04	4.06E-04	1.21E-03
Methyl isobutyl ketone	A	9.46E-05	4.00E-05	1.35E-04	1.16E-04	5.87E-05	1.74E-04
	B	-	-	-	-	-	
	C	1.62E-04	6.87E-05	2.31E-04	1.98E-04	1.01E-04	2.99E-04
	D	1.10E-04	4.65E-05	1.56E-04	1.34E-04	6.81E-05	2.02E-04
	E	1.89E-04	8.02E-05	2.70E-04	2.32E-04	1.18E-04	3.49E-04
	F	6.94E-05	2.94E-05	9.88E-04	8.48E-05	4.31E-05	1.28E-04
	G	1.21E-04	5.12E-05	1.72E-04	1.48E-04	7.49E-05	2.23E-04
	H	-	-	-	-	-	-
Hexane	A	-	-	-	-	-	-
	B	-	-	-	-	-	-

	C	-	-	-	-	-	-
	D	7.92E-04	3.35E-04	1.13E-03	9.68E-04	4.91E-04	1.46E-03
	E	8.89E-04	3.80E-04	1.28E-03	1.10E-03	5.57E-04	1.65E-03
	F	9.29E-04	3.93E-04	1.32E-03	1.14E-03	5.76E-04	1.71E-03
	G	4.40E-04	1.86E-04	6.26E-04	5.37E-04	2.73E-04	8.10E-04
	H	-	-	-	-	-	-
Acetaldehyde	A	1.09E-01	4.62E-02	1.55E-01	1.33E-01	6.77E-02	2.01E-01
	B	7.61E-02	3.22E-02	1.08E-01	9.30E-02	4.72E-02	1.40E-01
	C	1.35E-02	5.71E-03	1.92E-02	1.65E-02	8.36E-03	2.48E-02
	D	2.05E-01	8.66E-02	2.91E-01	2.50E-01	1.27E-01	3.77E-01
	E	2.86E-01	1.21E-01	4.07E-01	3.49E-01	1.77E-01	5.26E-01
	F	2.49E-01	1.05E-01	3.55E-01	3.04E-01	1.55E-01	4.59E-01
	G	1.60E-01	6.76E-02	2.27E-01	1.95E-01	9.91E-02	2.94E-01
	H	1.59E-01	6.72E-02	2.26E-01	1.94E-01	9.85E-02	2.92E-01
Propionaldehyde	A	1.24E-01	5.26E-02	1.77E-01	1.52E-01	7.71E-02	2.29E-01
	B	8.71E-02	3.69E-02	1.24E-01	1.06E-01	5.40E-02	1.60E-01
	C	1.23E-01	5.19E-02	1.75E-01	1.50E-01	7.61E-02	2.26E-01
	D	3.89E-02	1.65E-02	5.54E-02	4.76E-02	2.41E-02	7.17E-02
	E	8.36E-02	3.54E-02	1.19E-01	1.02E-01	5.19E-02	1.54E-01
	F	3.83E-02	1.62E-02	5.45E-02	4.68E-02	2.38E-02	7.05E-02
	G	4.09E-02	1.73E-02	5.82E-02	5.00E-02	2.54E-02	7.53E-02
	H	8.48E-02	3.59E-02	1.21E-01	1.04E-01	5.26E-02	1.56E-01
Ammonia	A	5.89E-03	2.49E-03	8.38E-03	7.19E-03	3.65E-03	1.08E-02
	B	4.78E-03	2.02E-03	6.80E-03	5.84E-03	2.96E-03	8.80E-03
	C	9.88E-03	4.18E-03	1.41E-02	1.21E-02	6.13E-03	1.82E-02
	D	6.87E-03	2.91E-03	9.78E-03	8.40E-03	4.26E-03	1.27E-02
	E	7.03E-03	2.98E-03	1.00E-02	8.59E-03	4.36E-03	1.30E-02
	F	2.56E-02	1.08E-02	3.65E-02	3.13E-02	1.59E-02	4.72E-02
	G	1.82E-02	7.72E-03	2.60E-02	2.23E-02	1.13E-02	3.36E-02
	H	4.69E-03	1.99E-03	6.68E-03	5.73E-03	2.91E-03	8.64E-03
Hydrogen sulfide	A	-	-	-	-	-	-
	B	-	-	-	-	-	-
	C	4.27E-03	1.81E-03	6.08E-03	5.22E-03	2.65E-03	7.87E-03
	D	-	-		-	-	-

	E	-	-		-	-	-
	F	4.72E-03	2.00E-03	6.72E-03	5.77E-03	2.93E-03	8.70E-03
	G	-	-		-	-	-
	H	-	-		-	-	-
